# A Pilot Study of Social Competence Assessment Using Interaction Rating Scale Advanced

**DOI:** 10.5402/2011/272913

**Published:** 2011-12-28

**Authors:** T. Anme, T. Watanabe, K. Tokutake, E. Tomisaki, Y. Mochizuki, E. Tanaka, B. Wu, M. Nanba, R. Shinohara, Y. Sugisawa

**Affiliations:** Graduate School of Comprehensive Human Sciences, University of Tsukuba, Tsukuba, Ibarnki 305-8574, Japan

## Abstract

*Purpose*. The purpose of this paper is to clarify the validity of the Interaction Rating Scale Advanced (IRSA) as an evidence-based practical index of social skills. *Methods*. The participants in our study were 17 high school students. The participants completed the five-minute interaction session and were observed using the IRSA. Their teacher evaluated their social competence based on regular observation in school. *Results*. The results indicated the high correlation between IRSA scores and teacher's practical evaluation. IRSA can measure social competence with high validity. *Conclusion*. The IRSA provides further evidence of the fact that in order to study social competence development, it is important to evaluate various features of the interaction like IRSA subscales.

## 1. Introduction

Social competence is determined by the complex interaction of the person themselves, their home and school environments, peer relationships, and the larger sociocultural environment [1]. Increasing numbers of school-aged children and adolescents with impulsive behavior and maladjustment to society requires society to prepare appropriate education and environments for those young people. Researchers, practitioners, and caregivers have been attracted to the study of social competence development for decades. Social competence is defined as the ability to understand others in the context of social interaction and to engage in smooth communication with them. Thus, social competence should be evaluated by the interaction between the person and social environment [2]. However, methodologies that consider persons in conjunction with their social environment across developmental stages have not yet been well developed.

Many researchers are focused on measuring the quality of a child's home environment and parenting, based on the theory that early rearing environment is significantly related to child development. Two instruments, namely, the Home Observation for Measurement of the Environment (HOME) [3] and the Index of Child Care Environment (ICCE) [4], are often used in research related to child development.

The HOME and the ICCE evaluate the children's rearing environment within natural settings, including the caregivers' emotional and verbal responsiveness to the child and the caregivers' acceptance of the child's behavior. The HOME has been adopted by studies conducted at the National Institute of Child Health and Human Development (NICHD) in the United States [5] and is also widely used in more than one hundred countries. The ICCE has been used to investigate the effect of child care on children's development in Japan [6]. In addition, the Mediated Learning Experience Rating Scale (MLERS) has been used to assess the sensitivity and teaching of adults (caregivers and teachers) toward children through observation of the adult-child interaction [7].

The tool that is currently used to assess social competence is the Social Skills Rating System (SSRS) [8] was used in the study conducted at the NICHD. The SSRS evaluates children's social competence on the basis of information provided by parents and teachers.

The factors of the construct of “social competence” have been discussed for years all around the globe. For example, SSRS [8], which has three factors, such as “cooperation”, “self-control”, and “assertion”, Caldarella and Merrell [9] mentioned five factors such as “peer relations”, “compliance”, “self-management”, “assertion”, and “academic”, while L. K. Elkskin and N. Elkskin [10] offers five factors such as “interpersonal”, “teacher-pleasing”, “self-related communication”, and “assertiveness”, and Kolb and Hanley-Maxwell [11] defines five factors including “peer and group interaction”, “problem-solving/decision-making”, “self-management”, “communication”, and “assertion”. All of them have common factors on “empathy/coordination”, “self-regulation”, and “assertion”. These three factors have been found to be stable over early child development from one to six years of age among Japanese children in a longitudinal study [12].

On the other hand, social competence evaluation of adults includes scales such as the Social Skills Inventory (SSI) [13] (six factors such as “emotional expressivity”, “emotional sensitivity”, “emotional control”, “social expressivity”, “social sensitivity”, and “social control”), ENDCOREs [14] (six factors such as “self-control”, “expressivity”, “sensitivity”, “assertiveness”, “responsiveness”, and “regulation”), Adult Behavior Checklist for Ages 18–59 (ASEBA) [15, 16] (six factors such as “adaptive functioning”, “empirically based syndromes”, “substance use”, “internalizing”, “externalizing” and “total problems”), and Weinberger Adjustment Inventory (WAI) [17] (six factors such as “distress”, “anxiety”, “depression”, “low self-esteem”, “low well-being”, “self-restraint”, “suppression of aggression”, “impulse control”, “responsibility”, and “consideration of others”).

The purpose of this study is to clarify the validity of Interaction Rating Scale Advanced (IRSA) as an evidence-based practical index of social competence.

## 2. Methods

### 2.1. Participants

The participants in our study were 17 high school students, 13 boys and 4 girls, 8 sixteen years old, and 9 seventeen years old.

In order to comply with the ethical standards, before conducting the research, all the participants signed informed consent forms and were made aware that they had the right to withdraw from the experiment at anytime. To maintain confidentiality of the personal information of the participants, a personal ID system was used to protect personal information. Further, all the image data were stored on a disk, which was password protected; only the researchers who were granted permission were given access to the data.

This study was approved by the ethics committee of the University of Tsukuba.

### 2.2. Measures

The IRSA is developed as an advanced version of IRS [18–20], which evaluate child-caregiver interaction by observation available under eight years of age. IRSA is used to measure social competence through five-minute observations of interactions. It includes 92 items that form a behavioral score and 6 subscales for an impression score, which are “self-control”, “expressivity”, “sensitivity”, “assertiveness”, “responsiveness”, and “regulation” (Table 1).

The total of 92 items was composed of several sources: original items by the study authors, several overlapping items from the IRS (Interaction Rating Scale) [18], the SSRS (Social Skills Rating Systems) [8], and the ENDCOREs [14].

Two different sets of variable are scored: behavior items and impression items for each subscale. Each subscale assesses the presence of behavior (1 = Yes, 0 = No), and the sum of all items in the subscale provides the overall behavior score.

Scores on the impression items and the overall impression item are on a five-point scale, where 1 = not evident at all, 2 = not clearly evident, 3 = neutral, 4 = evident, and 5 = evident at high level.

The evaluator completes the checklist composed of 92 items focusing on behavior (e.g., expresses his/her own feeling to the partner). Then the evaluator provides an impression on a 5-point scale of the level of interaction for each subscale.

Internal consistency of IRSA, as measured by Cronbach's alpha, is 0.84. The interobserver reliability was found to be 90%. IRSA can evaluate the interactions in a short period of time in daily situations.

Students' teacher evaluated their social competence using ENDCOREs [14] based on regular observation in school.

### 2.3. Procedure

In this study, the IRSA was evaluated as follows: a five-minute video recording of the setting of the interaction (two participants play game using “Keep it steady!”, which consists of the wooden ring, 6′′ long 27 sticks in vary widths, grab all the sticks together, slide the wooden ring around the center of the bundle, give it a twist, and stand it up. The game begins pulling out the piece, and taking turns back and forth until the structure collapses) was conducted. The recording was carried out in a room with two video cameras for each person. The dyads of participants were escorted into a room furnished with a small table and two chairs. The instructor introduced the game to both participants.

To score the behavior, two evaluators coded the participant's behaviors observed. The behavior during the interaction was coded as follows. If the participant displayed the behavior described in the item, a score of 1 was given; conversely, if the participant failed to display the behavior described in the item, a score of 0 was given. Total score was the sum of the score that participant received on all the subscales. A higher score indicated a higher level of social competence. The total IRSA score was the total score of the each subscale.

## 3. Results

**Figure 1:**
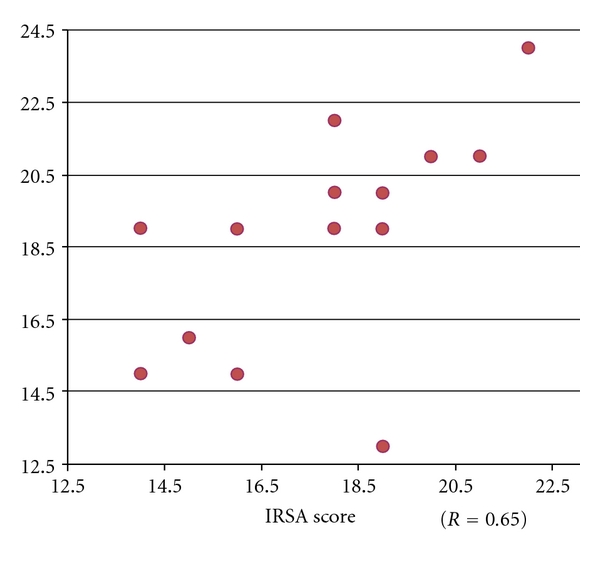
Correlation between IRSA and teacher's behavioral evaluation.

Figure 1 shows the correlation between the total score of IRSA and teacher's behavioral evaluation. It indicates a moderately high correlation (*r* = 0.65) between IRSA scores and teacher's behavioral evaluation.

 No significant gender and age differences were found on the subscales of IRSA.

## 4. Discussion

This study provides that IRSA can measure social competence with high validity. Social competence scale for child-caregiver interaction, named Interaction Rating Scale (IRS), was already found to be a reliable, valid, feasible, and practical tool for the studies of social interaction over time [18–20].

The strength of IRSA is three points as follow.

First of all, IRSA is easy to use in practice, because it has high adaptability for practice, since it can be used with the same subscales framework across ages.

Secondly, the IRSA can be used in international comparative studies, because it is based on the most common frameworks used all over the world. The subscales are based on various categories which are widely used in the research of social competence indicators.

Third, we have evidence of the IRSA in terms of construct and concurrent validity with the teacher's behavioral evaluation.

While the IRSA provides valuable insights, it is also important to acknowledge its limitations. First, this is just a small pilot study, only 17 participants in our study. We should use the results to generalize with cautiously. Second, the IRSA subscales might not cover all the dimensions of social competence, although we used the most common frameworks of social competence [21]. Third, while the IRSA expects to use same scoring standard as a standardized tool, different developmental features of items across developmental stages might be better to take into consideration [22]. Despite these limitations, the IRSA can be considered an established, valid screening instrument reflecting attributes of the social interaction. It provides further evidence of the fact that in order to study social competence development, it is important to evaluate various features of the interaction like IRSA subscales.

Further research has the potential to reveal the features of the social interaction development and enhance knowledge of implications for practitioners and caregivers.

## Figures and Tables

**Table 1:** Interaction rating scale advanced.

(i) Expressivity: Expresses his/her thoughts and feelings precisely	
(1) Vocalizes	
(2) Expresses his/her own feeling to the partner	
(3) Attempts to elicit help or consolation from the partner	
(4) Shows self-assertiveness to the partner through a gesture	
(5) Casts the partner a glance to seek sympathy	
(6) Shows the change of his/her feelings through facial expressions	
(7) Smiles or laughs	
(8) Attempts to make eye contact with the partner	
(9) Attempts to elicit a response from the partner	
(10) Looks at the partner's face to get information/clarification	
(11) Shows his/her feelings by words and actions together	

(ii) Assertiveness: States his/her opinion or position clearly to others	

(12) Speaks up to the partner about what he/she thinks	
(13) There are words and actions indicate his/her decision	
(14) Talks to, suggests, or lets the partner accomplish something while he/she gives the attention	
(15) Expresses his/her opinion to the partner	
(16) Verbalizes a differing opinion or position	
(17) Exhibits a differing opinion by his/her expression and gestures	
(18) Uses both verbal descriptions and non-verbal instruction	
(19) Provides guidance through explanation but not through order	
(20) Explains his/her opinion based on the level of competence/ability of the partner	
(21) Instructions and opinions are clear and unambiguous	
(22) Explains his/her opinion logically	
(23) Expresses his/her own idea after evidencing that he/she understands the partner's idea	
(24) Expresses his/her ideas after indicating his/her understanding to the partner through expression and gesture	
(25) Makes a decision after indicating that he/she understood the partner's idea/suggestion	
(26) Makes a decision after showing through non-verbal expression that he/she understood the partner	

(iii) Sensitivity: Ability to read the game partner's feelings and thoughts accurately	

(27) Shows an appropriate reaction by a change in expression and gesture	
(28) Vocalizes or speaks in response to the partner's verbalization	
(29) Vocalizes or adjusts own behavior in response to partner's verbalization	
(30) Looks at the partner or materials when he/she shows non-verbal behavior	
(31) Vocalizes in response to the partner's behavior or nonverbal cues	
(32) Babbles, makes a facial expression, or moves in response to the partner's behavior or a nonverbal cues	
(33) Vocalizes after noticing the changes in the facial expression of the partner	
(34) Looks at the partner or materials after noticing the changes in the facial expression of the partner	
(35) Vocalizes, expresses, or moves based on the change in partner's expression	
(36) Smiles or frowns within five seconds after the partner's vocalization	
(37) Looks at the partner's face or eyes when the partner attempts eye contact	
(38) Behaves in appropriate response to the partner's gestures, or changes in expression	

(iv) Acceptance: Understands and respects the partner's opinion or position	

(39) Smiles in response to the partner's smile	
(40) Praises the partner's efforts, success, and behavior	
(41) Smiles, claps hands, or shows that he/she is glad when the partner is feeling happy	
(42) Shows empathy by verbal or non-verbal response when the partner is in a bad mood	
(43) Emits positive, sympathetic, or soothing verbalizations in answer to the partner's feeling	
(44) Responds to the partner's vocalizations with an affectionate verbal response	
(45) Smiles at partner's verbalization	
(46) Nods sweetly in response to partner's verbalizations and/or actions	
(47) Emits a soothing non-verbal response (i.e., pat, touch, rock) at the partner's successful or fails	
(48) Smiles and/or nods at the partner during the episode	
(49) Does not vocalize or interrupt the partner while he/she is speaking	
(50) Nods at partner's comment	
(51) Accepts the partner's opinion partially or totally by saying “let us do it or by acting in a manner consistent with the partner's suggestion	
(52) Accepts the partner's opinion even when his/her own opinion differs	
(53) Pauses when the partner starts to verbalize	
(54) Does disturb the partner	
(55) Allows the partner to decide what he/she wants to do	
(56) Praises partner's skills in the course of assignment	

(v) Regulation of interpersonal relationship: Works with the partner to develop a good relationship	

(57) Provides an environment free of distractions for the partner	
(58) Does not make negative comments to the partner	
(59) Does not make negative behavior to the partner	
(60) Affirms the partner with nods or other gestures	
(61) Laughs while they are looking at each other	
(62) Laughs while they are looking at the same thing	
(63) Moves in the same manner as the partner moves	
(64) Does not turn away from the assignment and pays close attention to the partner	
(65) Verbally praises the partner during the assignment	
(66) Praises the partner with applause	
(67) Talks to the partner positively or encouragingly during the assignment	
(68) Says “Thank you” to the partner when he/she grants a concession	
(69) Does not criticize the partner when they have differing opinions	
(70) Tries to talk with the partner logically when they have differing opinions	
(71) Tries to avoid emotional conflicts with the partner	
(72) Tries to respond calmly when the partner becomes angry or agitated	

(vi) Self-control: Ability to control personal emotions and behavior	

(73) Waits for the partner's reaction or action for at least five seconds	
(74) Emits appropriate movement of eyes	
(75) Emits appropriate phonation	
(76) Emits appropriate utterances	
(77) Emits appropriate movements	
(78) Makes clearly recognizable hand motions towards materials during the assignment	
(79) Concentrates on the task and is gentle with the materials	
(80) Does not interrupt partner's implementation	
(81) Is not destructive/rough with the materials	
(82) Not tense	
(83) Does not shout or raise his/her voice	
(84) Does not display distress cues even when the task does not go well	
(85) Is not rude to the partner	
(86) Tries not to displease the partner	
(87) Does not speak negatively of others	
(88) Does not curse at people or at things	
(89) Follows the rules of the game	
(90) Touches a task together	
(91) Emits appropriate emotional expression	
(92) Praises the partner when he/she succeeds, or when the partner fails he/she commiserates	
